# Foot Reflexotherapy Induces Analgesia in Elderly Individuals with Low Back Pain: A Randomized, Double-Blind, Controlled Pilot Study

**DOI:** 10.1155/2017/2378973

**Published:** 2017-11-29

**Authors:** Bruna Hoffmann de Oliveira, Anna Quialheiro de Abreu da Silva, Daniela Dero Ludtke, Fernanda Madeira, Graciela Mendonça da Silva Medeiros, Rodolfo Borges Parreira, Afonso Shiguemi Inoue Salgado, Luiz Augusto Oliveira Belmonte, Francisco José Cidral-Filho, Daniel F. Martins

**Affiliations:** ^1^Naturology Graduate Course, University of Southern Santa Catarina (UNISUL), Palhoça, SC, Brazil; ^2^Physiotherapy Graduate Course, University of Southern Santa Catarina (UNISUL), Palhoça, SC, Brazil; ^3^Postgraduate Program in Public Health, Federal University of Santa Catarina, Florianópolis, SC, Brazil; ^4^Experimental Neuroscience Laboratory, Postgraduate Program in Health Sciences, University of Southern Santa Catarina (UNISUL), Palhoça, SC, Brazil; ^5^School of Postural and Manual Therapy, Salgado Institute of Integral Health, Londrina, PR, Brazil

## Abstract

**Introduction:**

This study evaluated the effects of foot reflexotherapy on pain and postural balance in elderly individuals with low back pain.

**Design:**

Randomized, controlled pilot study.* Participants *(*n* = 20) were randomly assigned to 2 groups: individuals submitted to conventional foot massage (control group) or foot reflexotherapy (RT, intervention group) for a period of 5 weeks. Questionnaires on pain and disability (visual analogue scale [VAS] and Roland-Morris Disability Questionnaire [RMDQ]), heart rate variability, and orthostatic balance and baropodometric analysis were assessed at two intervals: before and after intervention.

**Results:**

RT group showed statistically significant differences when compared to control group in the following parameters: decrease in VAS scores for pain throughout the study, decrease in parasympathetic activity, and improvement in RMDQ scores. The two groups did not statistically differ in either orthostatic balance or baropodometric analyses.

**Conclusion:**

This study demonstrated that foot reflexotherapy induced analgesia but did not affect postural balance in elderly individuals with low back pain.

## 1. Introduction

Chronic pain is a public health problem that causes serious personal and social afflictions [1]. Chronic low back pain, for example, is the second leading cause of disability among American adults. Health expenditures for adults with spinal problems in the United States have been progressively increasing from the 1990s to 2000s, with a recent estimate of roughly $6,000 per patient per year [2].

Chronic pain is especially problematic as the elderly population continuously grows worldwide and as with the aging process degenerative and chronic diseases associated with pain become more common, in addition to the higher probability of falls and fractures due to increased postural imbalance [3, 4].

To keep one's balance, the postural system adjusts one's mass center in its stability limits through the integration of sensorial receptors (somatosensory, visual, and vestibular), the skeletal muscle system, and ambient feedback. Together, these factors keep the orthostatic posture of the body under the foot surface [5].

Physiological processes related to pain are also regulated by the action of the autonomic nervous system. Among the techniques used for its evaluation, heart rate variability has emerged as a simple and noninvasive measure of autonomic impulses, representing one of the most promising quantitative markers of sympathetic and parasympathetic balance [6].

Clinical investigations have revealed that the most commonly used pharmacotherapies for the treatment of low back pain are opiates, nonsteroidal anti-inflammatory drugs, anticonvulsants, and antidepressants [7], although conventional pharmacotherapy may often be limited due to reoccurring collateral effects [8]. Consequently, the use of complementary and integrative (CI) approaches for chronic low back pain management may be of clinical relevance, especially if used prophylactically or in the management of treatment-related side effects. CI approaches may concurrently help reduce health costs and improve the patient's quality of life [9, 10].

In fact, a recent study reported that 44% of primary care patients with chronic pain receiving opioid therapy had used CI treatments in the previous 12 months. Additional research examining the use of these therapies for various chronic pain states report rates ranging from 35% to 63%. Over two-thirds of the Americans with chronic back pain, more specifically, make use of CI therapies, and these numbers increase to 44% of the population with ages between 50 and 59 [11, 12].

As the use of CI therapies for the treatment of lower back pain continues to increase, a series of questions about treatment effectiveness, safety, and cost are still not completely elucidated [13]. Nevertheless, advances are continuously being made by the National Center for Integrative and Complementary Health (NCCIH), which since 1999 has been gradually answering these questions for many CI therapies, including reflexotherapy [14].

Reflexotherapy is a well-known CI practice; in Norway and the United Kingdom, for instance, reflexotherapy is the most popular form of complementary therapy [13]. “Reflex,” in the sense of the word, means the “reflection or projection” of organs, systems, and structures of the body onto the feet (or hands) of the patient. Based on this premise, a trained therapist could positively influence overall bodily functions by simply “massaging or stimulating” such “projections.” The well-known Auriculotherapy is also based on similar principles [15].

Despite its popularity, only a few meta-analyses evaluating the effects of reflexotherapy have been conducted [16–18]. Therefore, additional studies on the effects of this therapy are needed.

Given the relevance of studies with CI therapies in the elderly population and the limited amount of literature on the efficacy of foot reflexotherapy for the treatment of lower back pain, the present research investigated the effects of a foot reflexotherapy protocol on low back pain and orthostatic balance in elderly individuals.

## 2. Materials and Methods

### 2.1. Participants

This is a randomized, double-blind, controlled pilot study with 20 elderly individuals previously diagnosed with chronic nonradicular low back pain. As it was a pilot study, a convenience sample was used and the elderly belonging to the Institutional Program “Grandpa in Action” of UNISUL were recruited. The participants were randomly assigned to 2 groups and submitted to either conventional foot massage (to serve as control group) or foot reflexotherapy (RT, intervention group) for 5 weeks. Randomization was performed using sealed envelopes after the assessments. Participants undergoing drug therapy followed their individual treatments throughout the study period.

### 2.2. Procedures

The study was approved by the Ethics Committee for Research Involving Human Beings of UNISUL (protocol number CAEE: 19237513.7.0000.5369). Participants were informed of the procedures and objectives of the study and signed an informed consent document. All evaluations and treatments were conducted at the Laboratory of Pain and Movement Studies (LaDeM) at UNISUL, Unidade Pedra Branca do Campus Grande Florianópolis, Santa Catarina, from 1 September to 31 October 2013.

### 2.3. Research Design

**Figure 1:**
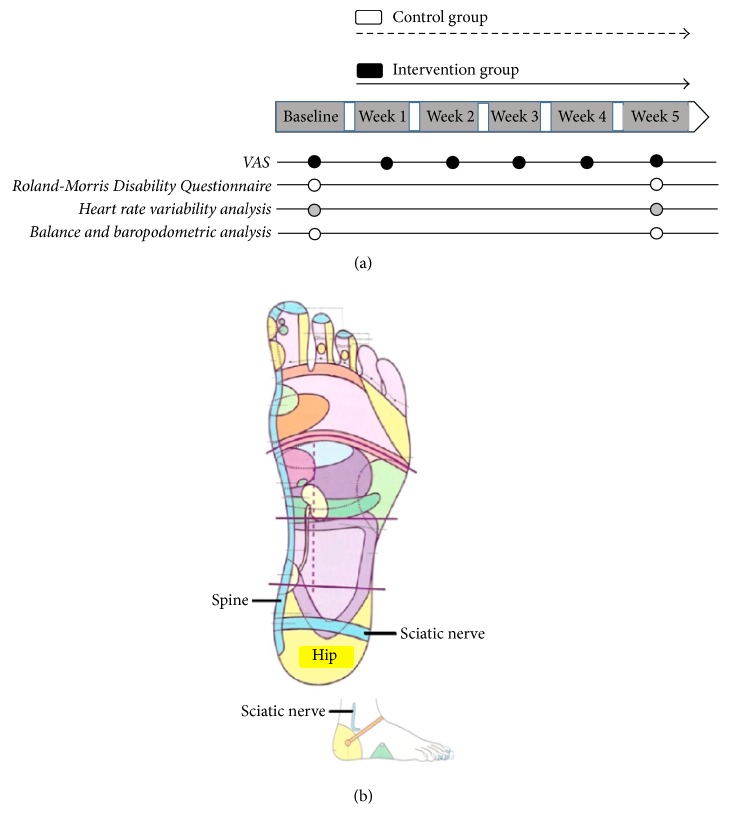
Study outline.

Baseline and final assessments were performed on Mondays. The final evaluations were performed after 5 sessions. At first, anthropometric measurements and vital signs were evaluated for all participants, followed by visual analogue scale (VAS), Roland-Morris Disability Questionnaire, heart rate variability analysis, and postural balance and baropodometric analysis (Figure 1(a)).

The evaluations were performed in a double-blind manner, in which neither the patients nor the researcher knew who was receiving the treatment and who was in control.

Inclusion criteria were as follows: (1) being over 60 years of age and (2) having a medical diagnosis of unspecified low back pain. Exclusion criteria were as follows: (1) presence of malignant diseases such as cancer and degenerative diseases, (2) presence of hypertension or labyrinthitis, or (3) presence of lesions in the feet, such as fissures, fistulas, dermatitis, or any damage to the integrity of the skin.

### 2.4. Intervention Protocol

RT group (*n* = 10) underwent a standardized and previously established protocol for the relief of low back pain as described by Gillanders [19] which included the “reflex” areas of the spine, hip, and primary and secondary sciatic nerve areas (Figure 1(b)). Each movement in these areas was repeated eight times, using as reference Gillanders Map (2008) [19], as shown in Figure 1(b).

Control group or conventional massage therapy (*n* = 10) received foot massage with kneading and sliding movements consisting of large movements that were applied in the same areas of the feet as those used in RT group to rule out the possible effect of muscle stimulation [20].

The groups received a total of 5 20-minute sessions. Neutral cream (VitaDerm®) was used in both approaches.

### 2.5. Measures

#### 2.5.1. Pain and Functional Capacity Evaluations

Pain assessment was performed with the visual analogue scale (VAS) which consists of a horizontal line with a length of 10 centimeters, numbered 0 to 10, marked “NO PAIN” at one end and “MAXIMUM PAIN” at the other. The participants mark the point that represents the intensity of their pain [21].

#### 2.5.2. Roland-Morris Disability Questionnaire (RMDQ)

The Roland-Morris questionnaire, which tests the individuals' disability, was performed only before and at the end of the study. The questionnaire consists of 24 statements that involve the functionality and limitations that low back pain can cause in everyday situations. The level of improvement of participants is calculated based on their initial score, allowing an evaluation of the individual evolution [22]. The RMDQ has 24 items with scores ranging from zero to one (yes or no) and a total that ranges from zero (no disability) to 24 (severe disability). For the calculation of the questionnaire score, the formula (final score − initial score/initial score)/100 is used and represents the percentage of clinical improvement [22]. In this study, we have used the version validated for use in Brazil [23].

#### 2.5.3. Heart Rate Variability (HRV) Analyses

Autonomic nervous system activity was performed using heart rate variability (HRV) with the Nerve-Express® software (Heart Rhythm Instruments, Metuchen, NJ, USA). Data acquisition was conducted by means of a transmitter belt (Polar® T31 coded™ Transmitter, Electro Oy, Finland), placed on the chest of the participant over the xiphoid process line, and a waist-strapped heart rate receptor coupled to a computer for processing and storage of captured data.

Each participant started the test in dorsal decubitus on a treatment table and after 192 R-R intervals of the heartbeat, the participant was instructed to stand up and face forward remaining still until the end of data acquisition, which is 448 R-R intervals.

All evaluations were conducted in a controlled environment (light, temperature, and sound). The HRV signals were compiled with MATLAB routines (The MathWorks, Natick, MA) and analyzed in time and frequency domains using Kubios HRV software version 1.1 (Biosignal Analysis and Medical Imaging Group, Kuopio, Finland). The frequency ranges analyzed in this study are in accordance with previous guidelines published by the American Heart Association [24], which indicate the recommendations and interpretations for the measurement of HRV.

The variables used for this study in the time domain were obtained by determining the corresponding RR intervals at any point in time [6, 24]. The variables analyzed were the mean RR (mean values of NN intervals from a time period), heart rate (beats/min) intervals, SDNN (standard deviation of NN intervals, a total estimate of HRV), and rMSSD (the square root of the mean squared differences of successive R-R intervals, a parasympathetic marker). These variables are routinely measured during the supine position.

The frequency domain variables measured in this study (contributing to the understanding of the autonomous fluctuations of the RR intervals in the heart rate register) were measured in both supine and orthostatic positions. The variables analyzed were low frequency (LF, modulated by both parasympathetic and sympathetic activities) and high frequency (HF, modulated exclusively by parasympathetic activities), both in absolute values (ms^2^) and in normalized units (nu).

#### 2.5.4. Assessment of Orthostatic Balance

To evaluate orthostatic balance, a pressure platform (Medicapteurs, S-PLATE model; Balma, France) connected to a computer was used. Body oscillations were recorded in a frequency of 10 Hz. The platform has the following characteristics: 610 mm wide, 580 mm deep, 4 mm thick, and weighing 6.5 kg. The platform is equipped with 1600 pressure piezoelectric sensors (48 × 48) and acquires 100 images per second. Before each evaluation, the calibration of the platform was conducted using the individuals' weight. For the analyses, the participants were asked to remove their shoes and stand on the platform with the arms relaxed alongside the body for 30 seconds. The participants were instructed to look at a fixed point straight ahead and avoid head movements. In an additional round of data acquisition, the participants were asked to keep their eyes closed (which may influence orthostatic balance). The distance between participants' feet was standardized as the normal opening for each individual to reproduce a natural and comfortable position.

The procedures for measuring the distribution of static plantar pressure were the same as those performed in the stability analysis. Peak pressure was described by the average found in the 30 seconds of acquisition of the measurements and expressed by foot area. The areas were defined according to the following: (a) the forefoot (the metatarsal heads and the toes) and (b) the hindfoot (the calcaneus region, the distal third of the foot). The right/left foot ratio was calculated as relative percentages and used for the analysis.

### 2.6. Statistical Analysis

The results were analyzed in the GraphPad Prism program (version 6.0, La Jolla, California, USA). Initially, the Shapiro-Wilk normality test was applied to evaluate the normality of the data. In the comparisons between preacquisition and postacquisition, the paired Student's *t*-test was used for the parametric data and the Wilcoxon test for the nonparametric data. When comparing control group to RT group in a single condition (before or after), the unpaired Student's *t*-test was used for the parametric data and the Mann-Whitney test for the nonparametric data. A 2-factor ANOVA followed by Bonferroni post hoc test was applied to determine statistical differences induced by RT or by time. Data were presented as mean ± standard deviation. Values of *p* < 0.05 were considered as statistically significant.

## 3. Results

**Figure 2:**
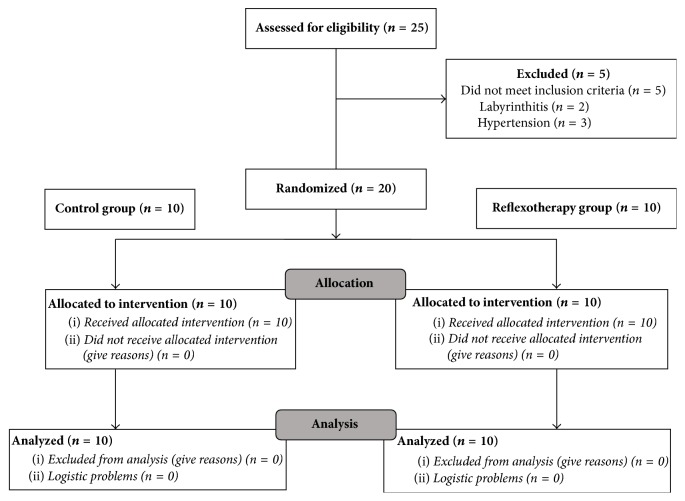
Group assignment.

A total of 25 participants with clinical diagnosis of low back pain were screened for the study. Of these, 5 met the exclusion criteria. Therefore, 20 participants were randomized to either group and all completed the study protocol, as shown in Figure 2.

In the baseline assessment, anthropometric variables showed no significant differences between the groups. The RT group and the control group had a mean of 60.7 ± 1.63 years and 60 ± 1.13 years (*p* = 0.43), respectively. The RT group consisted of three males and seven females and the control group consisted of four males and six females. The body mass varied between 68.3 ± 8.69 kg (RT group) and 72.4 ± 14.27 kg (control group) (*p* = 0.44). The height of the participants ranged between 161.9 ± 5.36 cm (RT group) and 159.5 ± 8.37 cm (control group) (*p* = 0.45). The body mass index varied between 25.67 ± 2.67 (control group) and 28.45 ± 5.39 (RT group) (*p* = 0.16).

**Figure 3:**
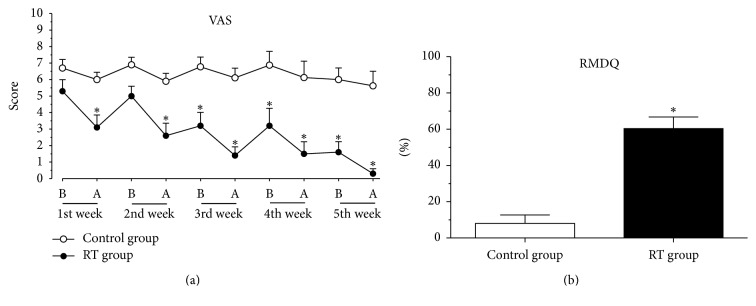
Effect of RT on (a) VAS and (b) RMDQ. Each group represents the mean of 10 participants, and the vertical lines indicate the mean ± standard deviation. ^*∗*^*p* < 0.05 when comparing RT group to control group.

Figure 3(a) illustrates the results regarding the evaluation of the effect of RT on back pain measured by the VAS. In the evaluation of the first day, there was no difference of the means; soon after RT or massage therapy, the values of the means were 3.1 and 6, respectively. On the second day of treatment, the mean values for the low back pain scores were not statistically different, but after the treatment there was a reduction in pain level in the RT group (2.6) when compared to the control group (6.9). On the third day of treatment, the RT group presented a mean of 3.2 and after the session the mean was 1.4. Values did not differ in the fourth week but were as low as 0.3 after the fifth reflexotherapy session.

Results presented in Figure 3(b) show that the average score obtained in the Roland-Morris Disability Questionnaire was 8.1% in the control group and that the mean score obtained in the RT group was 60.4%; this demonstrates that the RT group presented a 52.3% improvement in functional capacity at the end of the study.

**Figure 4:**
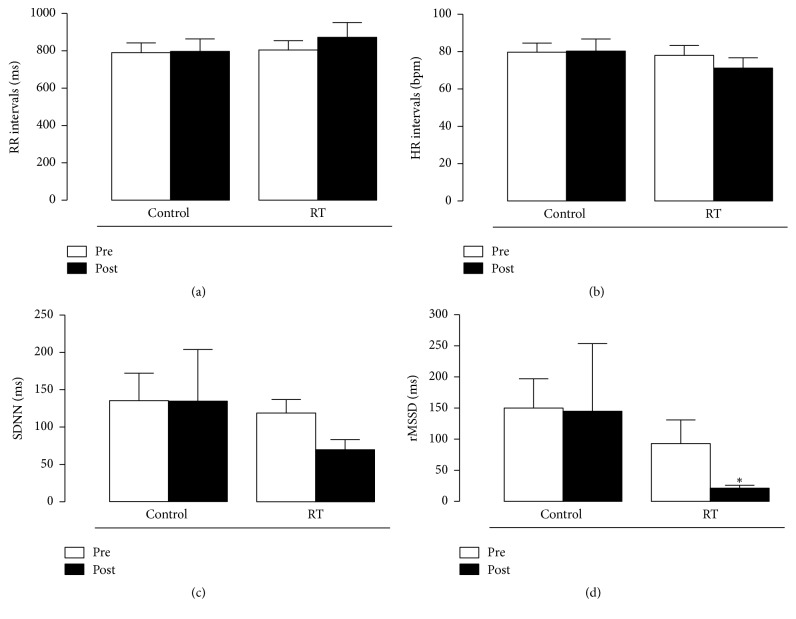
Effect of RT on (a) RR intervals, (b) HR intervals, (c) SDNN, and (d) rMSSD. Each group represents the mean of 10 participants, and the vertical lines indicate the mean ± standard deviation. ^*∗*^*p* < 0.05 when comparing RT group to control group.

**Figure 5:**
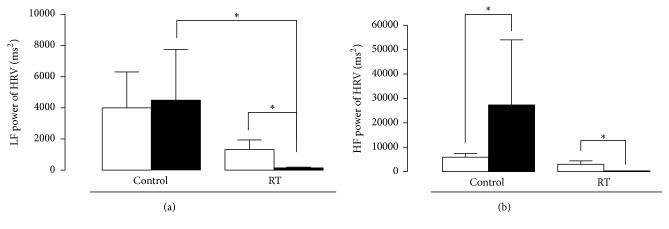
Effect of RT on (a) LF power and (b) HF power. Each group represents the mean of 10 participants, and the vertical lines indicate the mean ± standard deviation. ^*∗*^*p* < 0.05 when comparing RT group to control group.

The components of heart rate variability in the time and frequency domain are shown in Figures 4 and 5, respectively. In the time domain (Figure 4), only the rMSSD parameter showed a significant statistical difference before and after reflexotherapy sessions: from 93 ± 38 to 21.3 ± 4.7 ms (*p* < 0.01). RT group presented a greater reduction, after the session, when compared with control group (*p* = 0.01). The remaining time domain parameters did not statistically differ between the techniques (*p* > 0.05). RR interval means for massage therapy were 791 ± 116 before and 798 ± 147 ms after the session and in the RT group they were 805 ± 110 before and 873 ± 177 ms after the session (*p* > 0.05). Control group's heart rate was 79.7 ± 11 and 80.3 ± 14 bpm before and after the sessions, respectively. In the RT group, heart rate was 78 ± 12 before and 71 ± 12 bpm after the sessions. The SDNN parameter was 135 ± 82 and 135 ± 155 ms after massotherapy sessions. In the RT group, SDNN was 119 ± 41 before and 70 ± 30 after the sessions, almost reaching statistical difference (*p* = 0.06).

The parameters in the frequency domain (Figure 5) show varied results regarding the statistical differences between sessions and between groups. For absolute values in the LF parameter, control group was 3998 ± 2312 before the session and 4481 ± 3262 ms^2^ after the session (*p* > 0.05). However, in the RT group, HF values were 1324 ± 615 ms^2^ before and 154 ± 42.5 ms^2^ after the session (*p* < 0.001). Among the groups, a statistically significant reduction was observed.

After the session, RT group presented a significant decrease when compared with control group (*p* < 0.05), as well as for the LF parameter in normalized units (nu) (*p* < 0.001), but there was no difference (*p* > 0.05) before and after the techniques (control session = 37 ± 9.35 for 52 ± 12.4 and RT session = 46.7 ± 10.8 nu). For absolute HF values, there were statistically significant differences both within (*p* < 0.001) and between (*p* < 0.05) groups. For the control group, the value was 5921 ± 1523 before and 27372 ± 26685 ms^2^ after the session. For the RT group, results were 3030 ± 1388 before and 76.2 ± 15.3 ms^2^ after the session. In normalized units, there was only difference between the groups after the session (*p* < 0.05): before the massage session, the value was 62.7 ± 9.3 and it was 48 ± 12.4 afterwards. In the RT group, the value was 53 ± 10.6 before and 38 ± 12.5 after the session.

Table 1 shows that there were no statistically significant changes in the values of the parameters related to the stabilometric analysis (sway velocity L/L and sway velocity A/P) of the participants.

In addition, no statistically significant changes were detected in the baropodometric analysis (right and left pressure and peaks) (Table 2).

## 4. Discussion

The results of the present study demonstrate that RT reduces lower back pain and increases functional capacity. Pain evaluation was performed using scientifically validated methods such as VAS, as well as the RMDQ, both world-known evaluation tools for back pain [21–23].

Interestingly, it was observed that in the first two sessions the analgesic effect produced by RT did not persist for the whole week. However, from the third session on, the analgesic effect of RT was prolonged, persisting for more than one week, an effect that was observed until the last treatment session.

Data from the RMDQ show an improvement of more than 50% in the RT group when compared to control group, which indicates that RT contributes to reducing the functional limitations caused by low back pain which usually prevent or disrupt the activities in the elderly population.

To promote better care for the elderly population, it is necessary to consider all the changes inherent to this age group: aging of organs and systems and functional impairment, often due to the presence of pain, which ends up becoming chronic [25] and causing older people a greater chance of falls and fractures due to postural imbalances [3, 4]. Among the various conditions of chronic pain, lower back pain is the second most common reason for medical appointments, second only to headache complaints [26].

Recently, several scientific studies have been carried out to assess the best approach for low back pain relief. These studies culminated in the development of various aspects that should be considered during treatment [27–29]. Therapies for low back pain should be directed towards pain relief, increased functional capacity, and delayed disease progression [27]. In this sense, this study evaluated the influence of an RT protocol upon back pain and orthostatic balance in elderly individuals.

The physiological processes of pain are also regulated by the action of the autonomic nervous system. Among the techniques used for its evaluation, heart rate variability has emerged as a simple and noninvasive measure of autonomic impulses, representing one of the most promising quantitative markers of sympathetic and parasympathetic balance [30].

An explanation for the decrease in parasympathetic activity along with increased analgesia may be related to attention levels, stimulated by reflexotherapy differently from the relaxing stimulus of massage therapy. Zavarize et al. [31] proposed that virtual games may stimulate the frontal cortex, which together with other structures act as modulators of pain perception, acting in motivation, planning, and creativity. This is contrary to the effect commonly observed in cardiac autonomic modulation, in which the reduction of pain is related to the increase of the cardiac parasympathetic component.

As the participants of this study were a group of active elderly individuals, the reduced parasympathetic activity may be the result of rebalancing of the autonomic nervous system, since the physical activities practiced by this group may have increased these parameters excessively [32].

The responses of the LF and HF components as seen in Figure 5 may be due to the manual pressure exerted during the sessions. Diego and Field [33] reported that, depending on the pressure exerted during a massage session, there may be an increase in sympathetic activity (LF) with increased light pressure, whereas with moderate pressure there is an increased parasympathetic activity (HF). Guan et al. [34] observed this in children admitted to a children's hospital as they were more agitated (increased LF) during the massage sessions. Xue et al. [35] showed that foot massage reduced anxiety and pain in women after a cesarean section, with decreased LF and increased HF. In the present study, in the RT group, there was a significant decrease of the LF component.

The mechanisms of action of foot RT are not yet elucidated; however, beneficial results with different protocols have been consistently achieved [10, 11, 13, 16–18, 36]. One theory is that the practice of reflexotherapy works in ways other than massage therapy and may cause a cumulative analgesic effect over time [17, 18, 20, 37].

The present study's interest in assessing the balance of elderly participants with chronic low back pain comes from recent studies that suggest the measurement of center of pressure (COP) oscillation for clinical follow-up on pain variables. The study by Ruhe et al. [3, 4] demonstrated this relationship and argued that pain may cause an increase in the presynaptic inhibition of muscle afferents, affecting central modulation of the muscle spindles generating prolonged latencies. These changes may lead to decreased muscle control and, as a result, increased postural oscillation.

Based on these recent studies that showed a positive relationship between chronic pain intensity and postural control [3, 4, 36], the second objective of the present study was to evaluate the orthostatic or postural balance of these elderly participants with low back pain. The results demonstrate that there was no statistically significant difference in baropodometric and stabilometric parameters assessed between the groups. In this regard, the results of this study are contradictory to those presented in the current literature [3, 4].

Taken together, these results suggest that the RT protocol used in the present research has specific analgesic effect because it only causes pain relief [16–18, 36], a fact that coincides with those found in the literature: In a study with 40 people with lumbosacral disc hernia, 62.5% of the people reported a reduction in pain. However, there is an immediate positive effect of RT for cancer patients who reported pain [10, 11].

In conclusion, the present study demonstrated that foot reflexotherapy induced analgesia but did not affect postural balance in elderly individuals with low back pain. Additional research to elucidate the underlying mechanisms behind this effect may prove very promising.

## Figures and Tables

**Table 1:** Orthostatic balance and stabilometric table results before and after intervention in control group and RT group.

Variables		Control group		RT group	
		OE	CE	OE	CE
Sway velocity L/L (mm/s)	Before	0,48 ± 0,15	0,95 ± 0,29	0,24 ± 0,07	00,89 ± 0,22
	After	0,16 ± 0,05	0,95 ± 0,24	0,41 ± 0,13	11,15 ± 0,41
Sway velocity A/P (mm/s)	Before	0,99 ± 0,34	0,97 ± 0,45	0,75 ± 0,27	0,89 ± 0,22
	After	0,82 ± 0,17	1,0 ± 0,32	1,2 ± 0,63	1,09 ± 0,60

*Note*. Values are expressed as mean ± standard deviation. OE: open eyes; CE: closed eyes; mm/s: millimeters per second; RT: reflexotherapy; L/L: laterolateral; A/P: anteroposterior.

**Table 2:** Orthostatic balance and baropodometric table results before and after intervention in control group and RT group.

Variables		Control group		RT group	
		OE	CE	OE	CE
Pressure R/L (%)	Before	4,20 ± 3,04	5,20 ± 4,34	13,40 ± 11,70	11,38 ± 11,83
	After	7,33 ± 6,00	5,77 ± 7,31	9,00 ± 6,41	9,40 ± 6,73
Forefoot peaks L (area/cm^2^)	Before	633,3 ± 127,5	609,2 ± 111,5	626,5 ± 84,52	559,61 ± 103,5
	After	617,4 ± 128,7	612,1 ± 109,9	616,9 ± 145,1	660,37 ± 93,90
Hindfoot peaks L (area/cm^2^)	Before	905,2 ± 262,7	817,5 ± 121,3	814,6 ± 177,5	777,2 ± 178,8
	After	902,6 ± 124,2	851,4 ± 110,0	940,4 ± 150,8	888,4 ± 121,7
Forefoot peaks R (area/cm^2^)	Before	596,6 ± 141,8	587,1 ± 140,2	569,5 ± 210,5	555,5 ± 197,4
	After	709,2 ± 188,5	699,8 ± 163,2	528,9 ± 129,0	550,63 ± 95,91
Hindfoot Peaks R (area/cm^2^)	Before	962,8 ± 330,9	852,5 ± 145,9	761,6 ± 185,4	774,76 ± 160,9
	After	896,7 ± 178,4	859,9 ± 152,4	940,3 ± 189,3	888,77 ± 180,4

*Note*. Values are expressed as mean ± standard deviation, except for the R/L pressure, which is expressed in %. R: right; L: left; OE: open eyes; CE: closed eyes.
